# Supplementary material for: Causal interplay between lactose intolerance and gut microbiota: a combined bidirectional Mendelian randomization and in vivo validation study
Source: Front Nutr. 2026 Jun 1;13:1803337. doi: 10.3389/fnut.2026.1803337 (PMC13265576; doi:10.3389/fnut.2026.1803337)
Supplement: Supplementary file 1 [file Data_Sheet_1.zip › supplementary materials/Forward/leave one out cross validation/ebi-a-GCST90027451.finngen_R12_E4_LACTONAS.pdf]

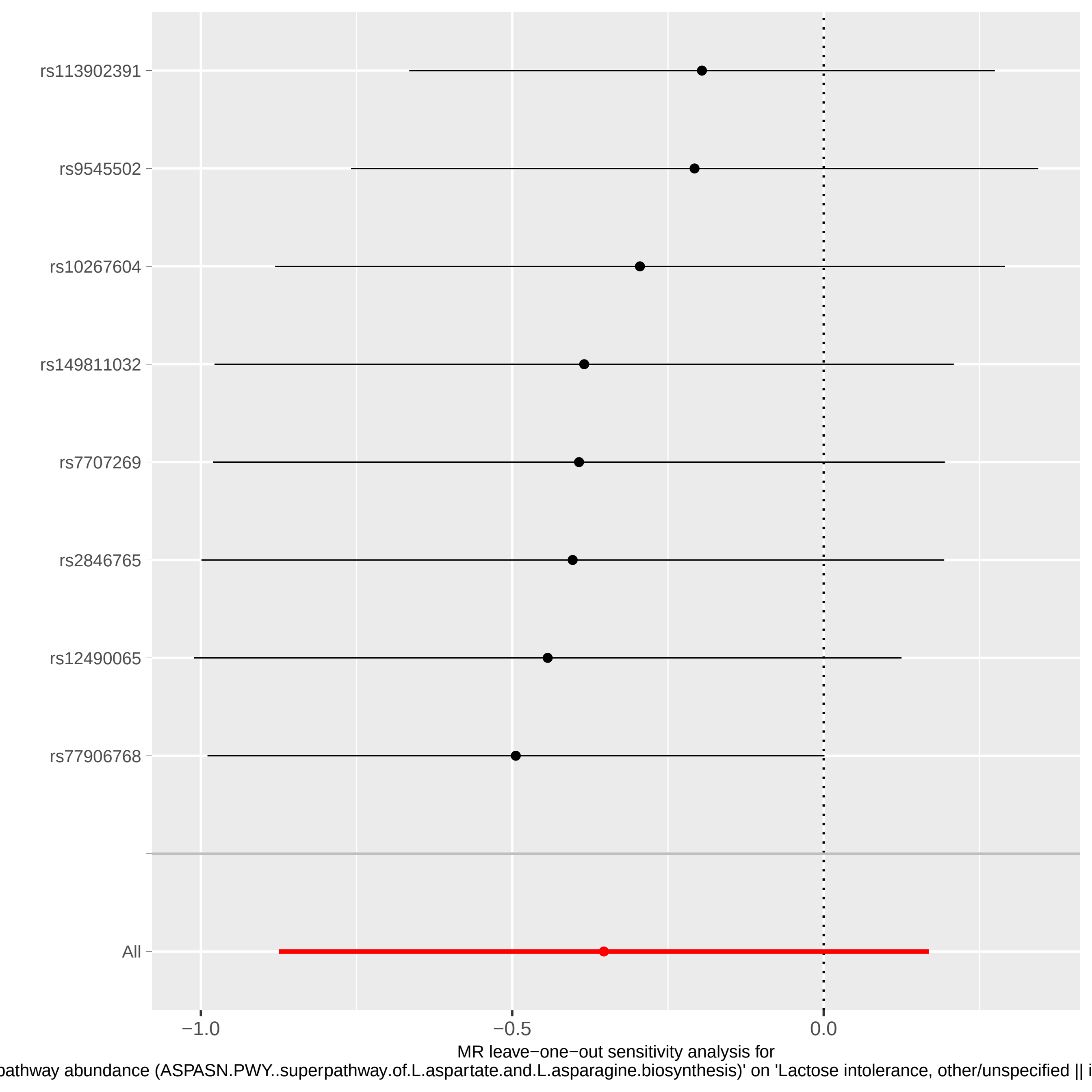

rs113902391
rs9545502
rs10267604
rs149811032
rs7707269
rs2846765
rs12490065
rs77906768
All
−1.0
−0.5
0.0
MR leave−one−out sensitivity analysis for
pathway abundance (ASPASN.PWY..superpathway.of.L.aspartate.and.L.asparagine.biosynthesis)' on 'Lactose intolerance, other/unspecified || i
